# Recapitulation of molecular regulators of nuclear motion during cell migration

**DOI:** 10.1080/19336918.2018.1506654

**Published:** 2018-09-27

**Authors:** Alexandra Sneider, Jungwon Hah, Denis Wirtz, Dong-Hwee Kim

**Affiliations:** a Department of Chemical and Biomolecular Engineering, Johns Hopkins University, Baltimore, MD, USA; b KU-KIST Graduate School of Converging Science and Technology, Korea University, Seoul, Republic of Korea

**Keywords:** Nuclear mechanics, Cell migration, LINC complex, Cytoskeleton

## Abstract

Cell migration is a highly orchestrated cellular event that involves physical interactions of diverse subcellular components. The nucleus as the largest and stiffest organelle in the cell not only maintains genetic functionality, but also actively changes its morphology and translocates through dynamic formation of nucleus-bound contractile stress fibers. Nuclear motion is an active and essential process for successful cell migration and nucleus self-repairs in response to compression and extension forces in complex cell microenvironment. This review recapitulates molecular regulators that are crucial for nuclear motility during cell migration and highlights recent advances in nuclear deformation-mediated rupture and repair processes in a migrating cell.

## Introduction

Cell migration is a hallmark of embryogenesis, wound healing, immune responses, and the progression of diverse human diseases including metastatic cancers [1],[2]. Accumulating evidence suggests that the rate-limiting step in cell migration through the extracellular matrix of connective tissues *in vivo* is the deformation of interphase nucleus [3,4]. The nucleus contains hierarchically structured nucleic acids and histone complexes that regulate cell functions by genetic and epigenetic mechanisms [5]. The interphase nucleus with viscoelastic solid properties [6,7] can elastically rebound following a mechanical deformation through multiple physical connections from the extracellular matrix across the plasma membrane to the nucleus.

While intracellular nuclear positioning, deformation, and motility were previously thought to be passively determined by cell movement [8], recent discoveries on molecular connections between the nuclear envelope and cytoskeleton suggest that the nucleus can actively change its shape [9,10] and repair the nuclear envelope to protect the nucleus [3,11]. More new evidences have revealed unprecedented active roles of nuclear dynamics during cell migration. For instance, cell polarization, the essential step to initiate cell migration [12], requires intracellular nuclear repositioning [10,13]. Indeed, nuclear mis-positioning and abnormal nuclear shaping are associated with the progression of diverse diseases such as cardiomyopathy [14,15] and autosomal recessive axonal neuropathy [16,17]. Even in three-dimensional (3D) cell migration, nuclear deformation is regarded as a critical rate-determining factor in migration of various cancer cells [18,19].

Therefore, molecular understanding of how the nucleus moves and responds to extracellular and intracellular mechanical stimuli caused by cell migration could provide a roadmap to find new molecular targets to develop effective therapies for human diseases. To this end, *in vitro* studies of mesenchymal cell migration have been attempted to recapitulate *in vivo* cell migration in conventional planar two-dimensional (2D) platforms or within cellular microenvironment-mimicking 3D extracellular matrices where cells dynamically present migration assisting cytoskeletal structures such as contractile lamellipodia, dendritic pseudopodial protrusions, and/or invadopodia [20–23].

3D cell migration operates under molecular pathways fundamentally different from 2D cell migration. Generation of mechanical forces necessary for net translocation results in dimension-specific differential cell motility [24,25]. For example, mechanical rigidity of the extracellular microenvironment can modulate prostate cancer cell migration differently between 2D and 3D environment. Cells are more motile in a less rigid 3D matrix but they tend to move faster in a more rigid 2D matrix [26]. Moreover, focal adhesion and wide lamella with filopodia are formed at the leading edge of the cell located in 2D space [2], while cells in the 3D matrix, in contrast, form thick protrusions rather than lamellipodia or filopodia for migration [21]. This distinct cell motility and corresponding protrusion dynamics are best illustrated by different dimension-specific roles of cytoskeleton regulating proteins [e.g., actin-related protein 2/3 (Arp2/3) complex and neural Wiskott-Aldrich syndrome protein (N-WASP)] and their relevant signaling pathways [e.g., endothelial growth factor (EGF) signaling].

To recapitulate active roles of the nucleus during cell migration, an extensive overview of molecular machinery involved in the alteration of nuclear morphology and motion during cell migration is needed. This review mainly focuses on the role of the nucleus in relatively slow migration of mesenchymal cells and in the invasion of metastatic cancer cells. We will discuss recent advances in our understanding of how cells deform, translocate, and rotate their nucleus as they move. Cytoplasmic molecular regulators that mediate nuclear movement in the migrating cell and molecular interactions between nucleus and cytoskeleton during nuclear motion are addressed along with physical interpretation of morphological alteration of the nucleus. The last section discusses rheological aspects of the nucleus in a migrating cell and challenges that cells face during migration, with a special focus on nuclear envelope deformation, lamina rupture and repair, intranuclear stress asymmetry, and chromosomal damage when migrating through 3D matrices. Pathological complications resulting from defects in cell migration are also highlighted.

### Cytoplasmic molecular regulators of nuclear movement

While dorsal actin cables can advance the nucleus from the back of the cell forward during nuclear translocation [27], the nucleus is located close to the centroid of the cell during nuclear rotation [10]. Since nuclear positioning is a highly orchestrated intracellular process, complex molecular machinery is involved in the nuclear membrane and intracellular cytoplasmic space [13]. Below is a description of the impact of molecular factors on nuclear motility, starting from those in the cytoskeleton that dynamically bind to the outer nuclear membrane (ONM) through nucleus-cytoskeletal connections such as linkers of nucleoskeleton and cytoskeleton (LINC) complexes. Next, the focus is on molecular regulators in the inner nuclear membrane (INM) and associated nuclear lamina and chromatin. An overview of these regulators is presented in Table 1.

**Table 1.** Nuclear molecules involved in cell migration.

Molecular Player	Location	Function	Literature
Refilin	Cytoplasm	A novel family of filamin-binding short-lived actin regulators that are involved in cellular phenotypic alterations such as epithelial-to-mesenchymal transition	[41,44]
		Refilin A: promotes the actin-binding filamin A (FLNA) to convert FLNA into an F-actin bundles	
		Refilin B: organizes a perinuclear actin cap	
Filamin	Cytoplasm	A downstream effector of the refilin proteins, coordinates the reorganization of perinuclear actin cytoskeleton and regulates nuclear motion	[45]
Formin	Cytoplasm or within the Nucleus	Involved in nuclear motion during 3D cell migration by modulating cell adhesion and polarization in 3D matrix	[47]
Cdc42	Cytoplasm	Involved in nuclear positioning	[2,55]
SUN-1,-2	INM	Proteins that bind to nuclear lamins are required to position the nucleus by recruiting Syne-1 and Syne-2 to promote centrosome-nucleus coupling	[61,70]
The Klarsicht-ANC-1-Syne-Homology (KASH) domain	ONM.	This component of the LINC is involved in the positioning of the nucleus in the cell	[68]
Nesprin-1,-2	ONM	Connected with the actin cytoskeleton	[135]
Nesprin-3	ONM	Moves the nucleus forward to create a pressure gradient in the cell, interacts directly with plectin, and establishes the linkage to the intermediate filaments	[76–78,138]
Nesprin-4	ONM	Binds to kinesin-1 and positions the MTOC and Golgi for migration	[79]
Lamin	Nucleoplasm	Fibrous proteins in a mesh network that connect to chromatin directly or indirectly, exhibits distinct viscoelastic properties to stabilize the nucleus	[38,57,87,88,112]
		Lamin A: contributes to cell invasion, stability, nuclear elasticity, resistance to mechanical stress, gene expression, and differentiation. Regulates viscous features of the nuclear lamina.	
		Lamin B: acts as an elastic component of the lamina and restores local deformation	

Nuclear motion could be regulated by the mechano-receptors in the cell surface. β1 integrin transfers the extracellular force stemming from contraction of type 1 collagen through regulating a PI3k/Akt pathway, which helps the nucleus to penetrate the narrow pore [28,29]. Actomyosin contraction regulated by Rho kinase further mediates focal adhesions (e.g., talin, vinculin, and FAK) through integrin and rear end retraction, which helps nuclear translocation during restricted cell migration. Ultimately, the nucleus is squeezed and pushed to the front edge by rear end actomyosin contraction [29,30].

Cytoskeletal proteins play a critical role in mediating nuclear movement. In particular, actin dynamics is essential for cell movement, contraction, phagocytosis, cytoplasmic division, and intracellular transport [31–34]. During cell migration in a 2D microenvironment, molecular connection between the nuclear envelope and actin cytoskeleton determines nuclear shape and movement through highly contractile actin stress fibers that drape around the nucleus. For instance, well characterized apical stress fibers (ASFs) [35,36] frequently termed as perinuclear actin cap allow the cell to maintain rapid, sustained, and directed migration [10,37,38] while the nucleus typically displays elongated shape and translocational motion without rotation [10,39]. Contrary to 2D cell migration where unique cytoskeletal architecture dominantly regulates nuclear motion, vertical asymmetry of the perinuclear region in a 3D space is not precisely determined, causing more complex actin polymerization-based cellular structures (e.g. membrane protrusions) to dominate nuclear motion [40].

During cell migration, actin regulating proteins control F-actin formation so that nuclear movement is coordinated. Refilin proteins including RefilinA and RefilinB are a novel family of filamin-binding short-lived actin regulators involved in cellular phenotypic alterations such as epithelial-to-mesenchymal transition (EMT) which makes cells to promote metastasis by decreasing nuclear stiffness that is induced by the loss of lamin A/C and allows nucleus to translocate to the foreign microenvironment with severe physical stress [18,41–43]. RefilinA promotes actin-binding filamin A (FLNA) to assemble F-actin bundles whereas RefilinB organizes a perinuclear actin cap [44]. As a downstream effector of refilin proteins, FLNA coordinates reorganization of the perinuclear actin cytoskeleton that regulates nuclear motion in 2D cell migration [45].

In 3D cell migration, Arp2/3 and WASP-family Verprolin-Homologous Protein2 (WAVE2) mediate nucleation of the perinuclear actin network, which disrupts perinuclear shell of lamin A/C [46]. Therefore, cells become more deformable to overcome physical limits and thus they can migrate in confined channel [46]. Non-muscle myosin II (NMII) also plays a critical role in nuclear motion in 3D cell migration by applying contractile force to the nucleus that enables cells to penetrate into their neighboring tiny pores formed by fibrous network of the extracellular matrix [48]. Since myosin-II is associated with formin that binds to barbed ends of actin filaments, formins are also involved in nuclear motion in 3D cell migration by modulating cell adhesion and polarization in 3D extracellular matrix [47].

Recent studies have shown that actin reorganization and the relative position of microtubule-organizing center (MTOC or centrosome) are both important for efficient nuclear movement [49,50]. While the precise position of MTOC depends on various factors (extracellular microenvironment, cell morphology, and a variety of intracellular molecular events such as chromosome pairing, retrograde actin flow, aggregation of adhesion molecules [13,51]), the relative position of the nucleus and centrosome largely determines cell polarity. The distance between MTOC and cell centroid tends to increase as cell polarization progresses [52–54].

Cell division control protein 42 homolog (Cdc42) and transmembrane actin-associated nuclear (TAN) lines are essential molecular factors involved in MTOC and nucleus positioning during cell polarization for directed migration [13]. The activity of Cdc42 is enhanced at the leading edge of migrating cells [54] where MTOC and Golgi apparatus are placed ahead of the nucleus [2,55]. This structural configuration stimulates microtubule formation that contributes to lamellipodia growth and vesicle delivery from Golgi to the frontal side of the cell by utilizing protrusion-mediating proteins [57]. For example, when fibroblasts experience shear stresses, small GTPase Cdc42 will localize the MTOC following the direction of flow [2,56]. Moreover, transmembrane actin-associated nuclear (TAN) lines (referred to as organization of nesprin-2 giant SUN2 and perinuclear actin cables) are known to induce nuclear rearward movement by retrograde actin flow to the nucleus [27]. Since retrograde actin flow is required for nuclear repositioning by exerting a pushing force to the nucleus through accumulation of intermediate filaments in front of the nucleus [50], TAN lines could also promote nucleus and centrosome orientation for nuclear forward movement [27,49,57,58].

This section described cytoskeletons and cytoplasmic regulators involved in nuclear positioning and movement during cell migration. Emphasis is placed on underlying cytoplasmic molecular mechanisms of 1) how actin filament and associated proteins could mediate nuclear motion, and 2) how MTOC and TAN lines could play a crucial role in locating the nucleus in a migrating cell.

### Nuclear-cytoskeletal connection in a migrating cell

**Figure 1.**
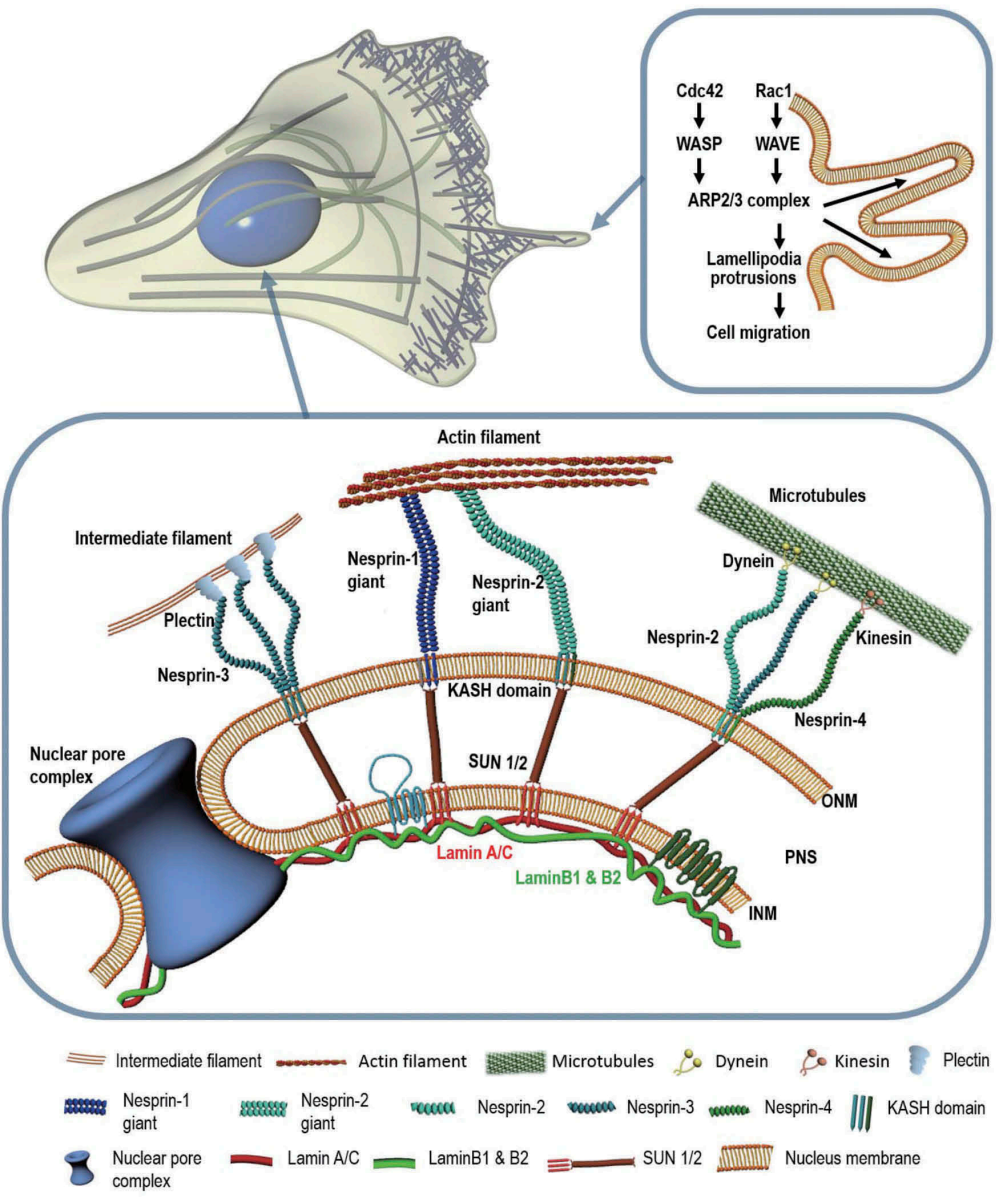
Molecular factors involved in cell migration. Schematic illustration showing key molecules involved in cell migration at the nuclear envelope and cell boundary. The highlighted signaling pathway depicts the formation of lamellipodia via Arp2/3 (**Right**). Cdc42: Cell division control protein 42 homolog; WASP: Wiskott-Aldrich syndrome protein; WAVE: WASP-family Verprolin-Homologous Protein; Arp2/3: Actin-related protein 2/3. The cytoskeleton is anchored to the intranuclear lamina through LINC molecular complexes located in the nuclear membranes (**Below**). ONM: Outer nuclear membrane; PNS: Perinuclear space; INM: Inner nuclear membrane; KASH: Klarsicht-ANC-1-Syne-Homolog; SUN: Sad1p; UNC-84.

This section focuses on how molecular regulators that interact with the nuclear membrane contribute to cell motility. While the mechanism by which cells interact with their nucleus for migration depends on tissue type, common proteins and signaling pathways are involved in this event [45]. Figure 1 illustrates the architecture of nuclear envelope that provides a framework of mechanotransduction for establishing mechanical interaction between nucleus and cell. Recently, identification of molecular components in the nuclear envelope and their connections with extranuclear cytoskeletons have revealed a force-induced molecular machinery that can alter nuclear morphology and movement during cell migration [3,10,11,38,59,60]. Linkers of nucleoskeleton and cytoskeleton (LINC) complexes, the most notable molecular structure interconnecting nucleus and cell, provide nuclear integrity and aid in cell migration by connecting cytoskeletal filaments to INM-associated proteins [61–64]. The key structure in LINC complex is the SUN-KASH bridge that has been covered by several comprehensive reviews [65–67]. What follows is a structural insight into molecular components of the SUN-KASH bridge and the underlying mechanism of how these components interact with cytoplasmic and nuclear domains.

SUN proteins are inner nuclear membrane proteins that interact with a Klarsicht-ANC-1-Syne-Homology (KASH) domain, a component of LINC complexes residing in the ONM [68]. The KASH domain found at the C-terminal of Nesprins (numbered 1 to 4) can bind to different proteins through specific cytoplasmic domains [69]. SUN proteins are required to position the nucleus by recruiting Syne-1 and Syne-2 to promote centrosome-nucleus coupling [61,70]. Nesprin proteins located in the ONM have various isoforms that mediate mechano-sensory functions via cytoskeletal connections [71,72]. Nesprin-1 and −2 bind to F-actin with calponin homology domains [73]. They also interact with microtubule motor proteins such as dynein and kinesin-1 [70,74]. Nesprin-2 is also associated with emerin, an inner nuclear membrane protein, and the common C-terminal region of lamin A/C [75]. Nesprin-3 that directly interacts with plectin is connected to intermediate filaments that are essential for directed cell migration [76,77]. Nesprin-3 dependent forward movement of the nucleus can create a pressure gradient in the cell, inducing 3D cell migration in a manner akin to a piston [78]. Nesprin-4 contributes to the relative positioning of the centrosome and nucleus in a migrating cell by binding to microtubule motor protein kinesin-1 [79].

The relative position of the nucleus in a migrating cell can change temporarily during repeated cycles of cell polarization which generally consist of stabilization of a main protrusion, formation of the leading edge, translocation of the cell body, and retraction of the rear end [80]. In the absence of physical confinement, cells preferentially migrate toward chemo-attractants, switching between two migration modes that display different nuclear morphologies. For instance, while persistently migrating motile cells typically show translocation of an elongated nucleus, slow and less motile cells exhibit rotation of a round nucleus [10]. This intracellular nuclear positioning is mediated by the cytoskeletal machinery such as microtubule motors and actin filaments that utilize LINC complexes [49]. Therefore, defects in LINC complex components are highly associated with the onset of pathological disorders [81] such as metastatic cancers, arthrogryposis multiplex congenita (AMC), and autosomal recessive autism that are largely attributed to mutations of nesprin and its binding partners [82]. Mutations in emerin also induce Emery-Dreifuss muscular dystrophy (EDMD) [83]. Ablation of both Nesprin-1 and −2 from myocardium results in cardiomyopathy and decreased response to biochemical signals [15]. Outer hair cells with Nesprin-4 mutations can inhibit cellular polarization, eventually inducing hearing impairment [84]. Diseases resulting from incomplete mechanotransduction by disruption of LINC complexes have been summarized in a previous review by Lammerding and Jaalouk [85].

LINC complex molecules are bound to nuclear lamina which consists of A-type (A/C) and B-type (B1, B2) lamins assembled with type IV intermediate filaments to form ~ 15 nm thick mesh network [86]. These lamins inside the nucleoplasm are connected to chromatin, exhibiting distinct viscoelastic properties to stabilize the nucleus [38,87]. A-type lamins contribute to cellular structural stability and dynamic response of the cell such as cell invasion, nuclear elasticity, resistance to mechanical stress, and cell differentiation by reinforcing the connection to the nuclear envelope [57,88]. In *in vivo* mimicking 3D microchannels, lamin A expression is diminished, causing the nucleus to become softer and deform more easily [43]. Moreover, nuclei with wild-type lamin is known to conserve the nuclear shape better than lamin A-deficient cells [89]. In the case of stem cell differentiation, while cell fate depends upon substrate compliance, lamin A expression is modulated by substrate stiffness [90]. Inside the nucleus, location-specific activated genes coincide with the organization of lamin A, demonstrating that A-type lamin also controls gene expression [38].

Collectively termed laminopathies are rare genetic disorders associated with defects of the nuclear envelope largely resulting from mutations of lamin-expressing *LMNA* gene. Laminopathic cells are typically accompanied by destabilized nucleus, increased fragility, and interrupted signaling [91]. In embryonic fibroblasts, for instance, lack of lamin A/C diminishes cellular responses to wounding, cell speed, cytoplasmic elasticity, and the linkage between MTOC and nuclear envelope [17], resulting in diverse rare genetic disorders such as muscular dystrophy, cardiomyopathy with conduction system disease, partial lipodystrophy, and progeria syndrome [88]. Therefore, understanding the molecular connectivity between nucleus and cell could lead to the development of new therapeutic strategies targeting nuclear motility and cell motion.

This section discusses how the LINC complex relays biophysical signals between the nucleus and cytoskeleton. The role of lamin proteins in constructing nuclear lamina in the INM and possible diseases associated with lamin deficiency are delineated.

### Mechanism of nuclear remodeling during cell migration

**Figure 2.**
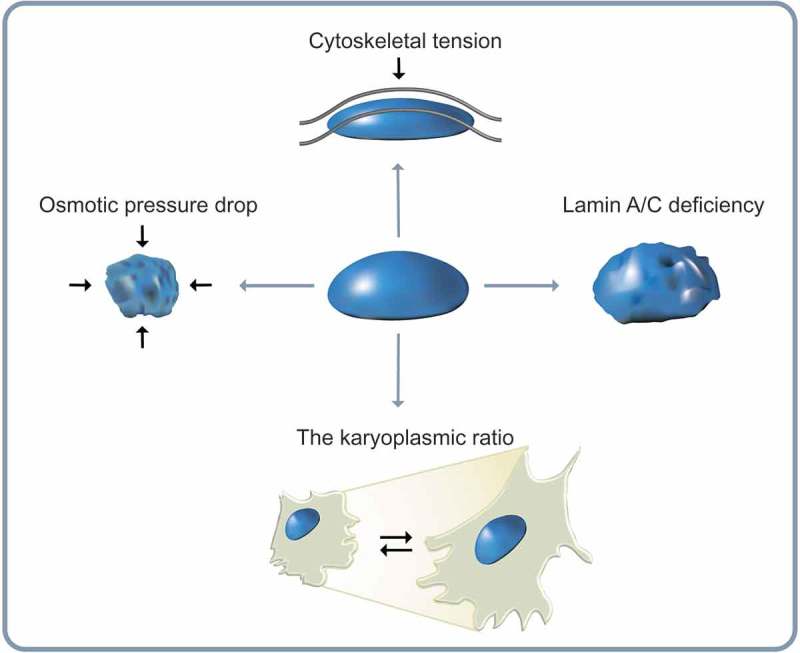
Alteration of nuclear morphology. Diverse morphological changes of the cell nucleus depend on the situation that the cell encounters. The nuclear volume decreases with reduction in osmotic pressure. The perinuclear actin cap aligns the nucleus along actin filaments during directed cell migration. A-type lamin deficiency attenuates nuclear structural integrity of human cells. The karyoplasmic ratio, a ratio of nuclear volume to cell volume (N/C ratio), is kept constant.

Nuclear remodeling involves structural deformation of the nucleus. The dimension of the nucleus such as shape and size is tightly regulated in the cell [92,93]. Nuclear morphology is one of the most important characteristic features in pathology. Abnormal nuclear morphology is routinely assessed in the clinic owing to its strong relevance to pathological alterations of cellular homeostasis, including cell migration, proliferation, and disease progression [94,95]. Nuclear volume can also be a promising determinant of normal nuclear mechanics involved in cell migration, e.g., nuclear compression and relaxation during cell migration through constricted channels or inside the 3D extracellular matrix [89,96,97]. The nucleus has reversible elastic behavior and plasticity to nuclear deformation [98]. Thus, alteration of nuclear shape and volume can have great impact on a cell’s ability to migrate through complex tissue environments. This section highlights molecular and biophysical mechanisms that regulate the response of the nucleus to mechanical stresses (Figure 2).

Nuclear shape is strongly dependent on intracellular and extracellular mechanical stimuli, including pressure difference across the nuclear membrane [96], cytoskeletal pre-stress [99], and topology of the nuclear envelope [100]. In higher eukaryotes, nuclear morphology is tightly regulated by nuclear lamina [101,102]. High resolution real time imaging [102] and micromanipulation of cell adhesion [39,104] have demonstrated that nuclear shape is systematically altered during cell migration through tight molecular interactions between the nuclear envelope and cytoskeletal components [10,89]. Nuclear motion during mesenchymal cell migration, for instance, typically features a repetition of a persistently migrating translocation and a hesitating less motile mode that precisely recapitulates the cycle of cell polarization [10].

Mechanotransduction, the essential relay of biophysical signals during cell migration, is mediated by molecular coupling between nuclear lamina and cytoskeletons. For example, perinuclear apical actin stress fibers specifically formed in the cell placed on 2D substrate exert vertical force onto the nucleus [96,105]. Accordingly, elevated nuclear pressure by the extranuclear actin stress fibers reorganizes A-type lamins to concentrate on the apical side of the nucleus where the compressive force is applied. The pressurized nucleus can further drive lamin A/C toward a more condensed state (e.g., compact interlaced polymer networks) [38]. Moreover, formation of actin stress fibers accelerates the assembly of A-type lamins in cells cultured on rigid matrices [9] by inhibiting the affinity of tyrosine kinases that can phosphorylate A-type lamins to ultimately induce degradation of nuclear envelope architecture [90]. Vertical polarization of A-type lamin is therefore, the consequence of adaptive response of nuclear lamina to external physical stimuli conveyed from the extracellular microenvironment [105]. In addition to substrate rigidity, various mechanical stimuli from extracellular matrix control the property of nucleus. Curvature of surface that cells adhere to also determines nucleus shape and physical property. Concave surface lifts the cell up, while convex surface pushes the cell down [106] because the surface curvature differs the direction of actin cytoskeletal force. Consequently, the nuclear structure on the convex surface is compressed and remodels to flat nuclear morphology with enhanced expression of A type-lamins [106]. Recent studies also demonstrated that surface topology, modulated by the interspace of PLGA micropillar array, changes nuclear shape. They showed that deformed nucleus was rapidly recovered by actin cables around the nucleus. Therefore, these profound evidences demonstrate that the nucleus is a center of mechanotransduction by remodeling its intra- and extra- spaces [107]

The karyoplasmic ratio, a ratio of nuclear volume to cell volume, is known to roughly remain constant in diverse cell growth conditions, genetic variations, and various stages of the cell cycle that could affect cell dimension and DNA content [108]. Therefore, nuclear volume change largely follows cell volume change [109]. Recent studies have further demonstrated that nuclear volume is also determined by a combination of two physical forces applied to the nuclear surface during migration: (i) an osmotic pressure drop across the nuclear envelope, and (ii) hydrostatic pressure difference between cytoskeletal forces and mechanical resistance of the nuclear envelope [110]. The functional relationship between changes in nuclear volume and nuclear shape can be directly observed by monitoring reductions in nuclear volume induced by detachment of cells from their adhesive substrate [96]. Indeed, cell detachment can induce decrease in nuclear volume of up to 50%. It is typically accompanied by the formation of deep invaginations in the nuclear envelope which in turn induce highly irregular nuclear shape [96]. These shape changes are due to balance between cytoskeletal forces and osmotic pressure drop across the nuclear envelope [111] where small molecules can flow through nuclear pore complexes that are permeable to water molecules [112,113].

This section describes nuclear shape and size changes that underlie nuclear remodeling. A-type lamins determine nuclear shape change while A-type lamin-bound actin stress fibers can relay biophysical stimuli stemmed from extracellular microenvironments into the nucleus. Thus, nuclear volume is highly dependent on osmotic pressure drop across nuclear membrane as well as cytoskeletal forces mediated by nuclear lamina-cytoskeletal connection.

### The role of nuclear rheology in three-dimensional cell migration

During *in vivo* cell migration, cells continuously encounter situations that they should deform their nucleus to move through narrow spaces. This nuclear deformation is enabled by material property of the nucleus that is conventionally modeled as a viscoelastic gel. This chapter elucidates rheological behaviors of nuclear envelope that mediate 3D cell migration.

**Figure 3.**
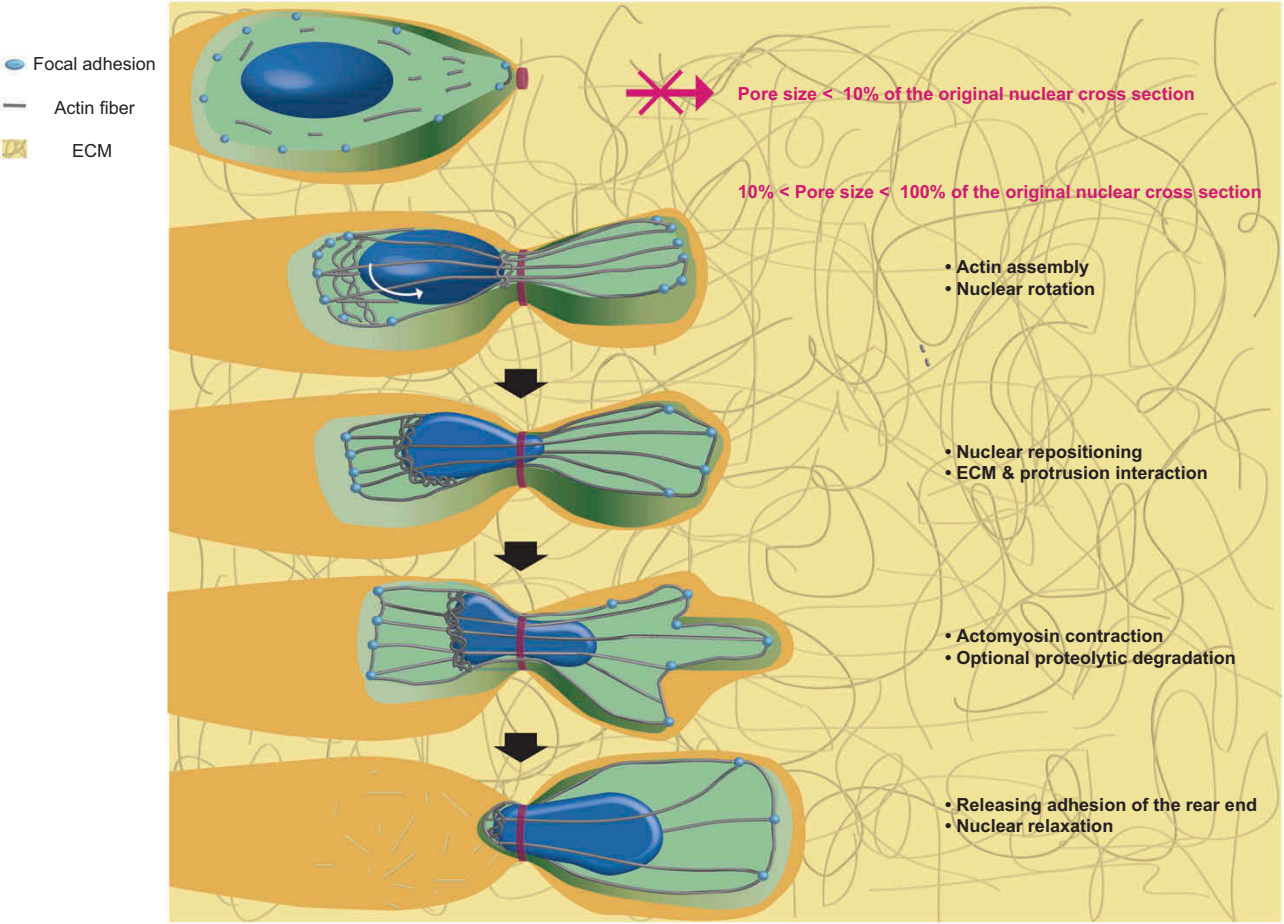
Nuclear deformation during cell migration through the extracellular matrix. Cell penetration into the extracellular matrix requires multiple steps: formation of a protrusion at the leading edge and nuclear rotation, nuclear repositioning and interaction with the ECM, myosin-dependent contraction, matrix remodeling, and finally release of adhesion force at the rear of the cell.

Intracellular organelles can also adapt to altered micro-environment that migrating cells experience to maintain their functions. One of these environmental challenges is confined space that cells encounter during their 3D migration inside tissues (Figure 3). Contrary to cell motion on planar 2D surface, 3D cell migration consists of five steps: (i) actin assembly, formation of protrusions, and nuclear rotation; (ii) sensation of ECM and intracellular nuclear repositioning; (iii) proteolytic degradation of ECM; (iv) myosin II-dependent posterior contraction; and (v) rear end releases [64]. As the nucleus is two- to ten-times stiffer than the cytoplasm [114], the nucleus must undergo deformation to fit through extracellular matrix pores and confined narrow channels formed by aligned muscle or nerve fibers [115].

Nuclear stiffness is not only affected by its intrinsic rheology (i.e., passively) that is mostly controlled by nuclear lamina and chromatin, but also governed by contractile cytoskeletal structure (i.e., actively) that is bound to the nuclear envelope and dynamically connected to the lamina through protein linkers [43,116]. A-type lamins (e.g., lamin A/C) known to raise nuclear stiffness [116,117] represent the viscous feature of nuclear lamina to relieve the mechanical force applied to the nucleus, ultimately making the nuclear lamina function as a ‘shock-absorber’ in various tissues. Meanwhile, B-type lamins (e.g., lamin B1 and lamin B2) mainly act as elastic components of the nuclear lamina. Therefore, the nucleus becomes more elastic when more mechanical forces are applied to restore local deformation along the nuclear surface [112,118]. Moreover, chromatin density affects the mechanical property of the nucleus. Chromatin density and nuclear stiffness are known to be increased when the nucleus is physically condensed [116]. Therefore, aggressively invading cancer cells display reduced chromatin condensation which induces nuclei to be more deformable [119].

During 3D cell migration, along with physical properties of the nucleus, nuclear dimension also mediates cell migration through complex 3D pores [3]. In general, pore sizes of less than 10% of non-deformed nuclear diameter can halt cell migration [114,120]. In combination with cell migration steps, recent studies have demonstrated that the rate-limiting step in 3D cell migration in the matrix is associated with rear end release that pushes cell forward. It is also closely related to deformation of lamin network and nuclear envelope as pore size becomes smaller [64,120,121]. This material property-dependent behavior is dominated by actomyosin contraction mediated by Rho-associated protein kinase (ROCK) which allows for sufficient intracellular tension to initiate cell migration through narrow pores [64,120]. Therefore, confined cell migration (e.g., cells located inside narrow micro-channels) require active acto-myosin contraction that cannot exist without A-type lamin [43].

Indeed, the role of lamin proteins in cell migration is not straightforward. It needs further investigation. For random cell migration in a 3D extracellular matrix, the nucleus requires A-type lamins to maintain its structural integrity to facilitate directed cell migration that requires nuclear deformation with the help of actin stress fibers [47,122]. In contrast, recent studies performed with microfluidic devices have suggested an unusual role of A-type lamin in 3D cell migration where microfluidic devices with a chemotactic gradient along micro-channels are devised to mimic the architecture of human connective tissues and monitor constricted cell migration *in vitro* [122,124]. It has been found that deficiency in A-type lamins can enhance the migrating ability of human fibrosarcoma and breast carcinoma cells so that they can move through constricted channels fast [43]. These results demonstrate that nuclear pressure is increased during confined cell movement along pores, resulting in lamina rupture, chromatin herniation, nuclear fragmentation, and breakage of double strand DNA [123,124]. Moreover, nuclear pressure-induced bleb formation along the nuclear envelope increases with reduced levels of lamin B1. Thus, lower level of lamin A/C and B2 increases the chance of nuclear rupture because A-type lamin prevents nuclear deformation [57]. Indeed, cells with lower levels of lamin A/C can migrate faster in confined micro-channels [3,124].

After constricted cell migration through 3um pore sized micro-channels, nuclear membrane rupture is induced [43,125]. Eventually, DNA repair factors (eg., Ku80, BRCA1 and RPA1) delocalize from intranuclear space to all around the cytoplasm and the nonlethal DNA damage occurrence (i.e., aneuploidy) was followed by the loss of DNA repair factors [126]. After entering the narrow pore, cells are elongated and microtubule-associated transcription factor GATA4 is upregulated, which mediates an endothelial-to-mesenchymal transition (EMT) [127]. Moreover, it is well established that cell and nuclear morphology switch intermittently between two distinct nuclear motion – nuclear rotation and translocation where one directional nuclear translocation is dominant in elongated cell shape (i.e., restricted cell migration through the microchannel) [10]. These results suggest that epigenetic changes are induced by the alteration of cell phenotype that is tightly regulated by nuclear motion.

This section highlights nuclear rheology regulating nuclear lamina, nucleus-cytoskeletal connection, and the nuclear envelope. This segment also provides the underlying mechanism of nuclear deformation during 3D cell migration that can result in epigenetic alterations associated with devastating human diseases.

## Conclusion and perspectives

This review explains how molecular regulators are involved in nuclear dynamics of migrating cells. Alteration of nuclear morphology is precisely tuned to preserve important cellular functions under mechanical stimuli such as shear and pressure-driven forces [3,128,129]. Nuclear dynamics is receiving growing attention from traditional cell biologists, biophysicists, as well as clinicians since diverse pathological processes are tightly regulated by nucleus-cell interaction during cell migration. The combination of nanotechnology and cell biology is actively applied to provide in-depth knowledge of nuclear behavior. For instance, traction force microscopy, a method developed and refined over the past twenty years, has enabled quantification and observation of mechanical interactions within the cell or between cells and their surrounding tissues [130,131], allowing for a better understanding of the role of the nucleus in a migrating cell. Microfluidic devices have also been improved to allow for systems that can better mimic 3D *in vivo* conditions [123]. While the field of the nuclear dynamics is quite new, diverse technical advancement has been made and this advancement aims for the achievement of both the imaging and quantification [10,54]. These methods have demonstrated that nucleus is a dynamic organelle that utilizes molecular connections between its lamina and the cytoskeleton (i.e., LINC complexes) which in turn assembles nucleus-linked actin fibers and generates mechanical forces to properly orient and drive directed cell migration [47,131]. Hence, it becomes more convincing that the nucleus is not passively dragged along within the migrating cell. Instead, it provides the necessary mechanical support to the cytoskeleton for efficient generation of contractile forces [133]. Recent studies on cardiac diseases such as myotonic dystrophy and osteoporosis have provided further support for the notion that the onset of some devastating diseases is attributed to defects of nuclear dynamics rather than genetic mutation itself [134,135]. Functional declines from defects in LINC complex are observed in aging process [136], and during cancer metastasis from the primary tumor site to remote tissue, physical properties of the nucleus act as a crucial factor [43]. Therefore, therapeutic target in nuclear motion is the nucleoskeletal LINC proteins and intranuclear factors affecting the nuclear property. Permanent or transient alterations of cell nucleus during cancer invasion or under the external mechanical stresses could be attributed to nuclear deformation in outer/inner nuclear membrane as well (e.g. fluctuation of lamin density and chromosomal defects). Consequently, nuclear morphology and molecular architecture reflect cells’ ability, and eventually predict disease progression of patients via intranuclear organizations. Additionally, the way to modulate the nuclear property (e.g. chromatin density, A-type lamin expression) toward the indicator of the healthiness is a new avenue for curing devastating diseases. Therefore, continued investigation of the structure and function of molecular factors within and surrounding the nucleus may allow clinicians to develop more efficacious therapies to treat a variety of diseases.
